# Childhood adiposity underlies numerous adult brain traits commonly attributed to midlife obesity

**DOI:** 10.1093/brain/awae198

**Published:** 2024-06-18

**Authors:** Scott T Chiesa, Lydia Rader, Victoria Garfield, Isabelle Foote, Sana Suri, George Davey Smith, Alun D Hughes, Tom G Richardson

**Affiliations:** Medical Research Council Unit for Lifelong Health and Ageing at UCL, Institute of Cardiovascular Science, UCL, London WC1E 7HB, UK; Institute for Behavioral Genetics, University of Colorado Boulder, Boulder, CO 80309, USA; Medical Research Council Unit for Lifelong Health and Ageing at UCL, Institute of Cardiovascular Science, UCL, London WC1E 7HB, UK; Institute for Behavioral Genetics, University of Colorado Boulder, Boulder, CO 80309, USA; Department of Psychiatry, University of Oxford, Oxford OX3 7JX, UK; Oxford Centre for Human Brain Activity, Wellcome Centre for Integrative Neuroimaging, University of Oxford, Oxford OX3 7JX, UK; MRC Integrative Epidemiology Unit, University of Bristol, Bristol BS8 2BN, UK; Population Health Sciences, Bristol Medical School, University of Bristol, Bristol BS8 1UD, UK; Medical Research Council Unit for Lifelong Health and Ageing at UCL, Institute of Cardiovascular Science, UCL, London WC1E 7HB, UK; MRC Integrative Epidemiology Unit, University of Bristol, Bristol BS8 2BN, UK

**Keywords:** life course Mendelian randomization, adiposity, obesity, neuroimaging, brain traits

## Abstract

Obese adults are often reported to have smaller brain volumes than their non-obese peers. Whether this represents evidence of accelerations in obesity-driven atrophy or is instead a legacy of developmental differences established earlier in the lifespan remains unclear.

This study investigated whether early-life differences in adiposity explain differences in numerous adult brain traits commonly attributed to mid-life obesity.

We used a two-sample life course Mendelian randomization study in 37 501 adults recruited to UK Biobank (UKB) imaging centres from 2014, with secondary analyses in 6996 children assessed in the Adolescent Brain Cognitive Development Study (ABCD) recruited from 2018. Exposures were genetic variants for childhood (266 variants) and adult (470 variants) adiposity derived from a genome-wide association study (GWAS) of 407 741 UKB participants. Primary outcomes were: adult total brain volume; grey matter volume, thickness and surface area; white matter volume and hyperintensities; and hippocampus, amygdala and thalamus volumes at mean age 55 in the UKB. Secondary outcomes were equivalent childhood measures collected at mean age 10 in ABCD.

In the UKB, individuals who were genetically predicted to have had higher levels of adiposity in childhood were found to have multiple smaller adult brain volumes relative to intracranial volume [e.g. *z*-score difference in normalized brain volume per category increase in adiposity—95% confidence interval (CI) = −0.20 (−0.28, −0.12); *P* = 4 × 10^−6^]. These effect sizes remained essentially unchanged after accounting for birthweight or current adult obesity in multivariable models, whereas most observed adult effects attenuated towards null [e.g. adult *z*-score (95% CI) for total volume = 0.06 (−0.05, 0.17); *P* = 0.3]. Observational analyses in ABCD showed a similar pattern of changes already present in those with a high body mass index by age 10 [*z*-score (95% CI) = −0.10 (−0.13, −0.07); *P* = 8 × 10^−13^], with follow-up genetic risk score analyses providing some evidence for a causal effect already at this early age. Sensitivity analyses revealed that many of these effects were likely due to the persistence of larger head sizes established in those who gained excess weight in childhood [childhood *z*-score (95% CI) for intracranial volume = 0.14 (0.05, 0.23); *P* = 0.002], rather than smaller brain sizes *per se*.

Our data suggest that the persistence of early-life developmental differences across the life course may underlie numerous neuroimaging traits commonly attributed to obesity-related atrophy in later life.

## Teaser

Using a genetic technique known as multivariable Mendelian randomization, Chiesa *et al.* show that differences in brain volumes that are commonly attributed to obesity in adults are likely to have instead arisen due to earlier obesity in childhood and then persisted across the lifespan.

## Introduction

Obesity represents one of the greatest challenges to human health and longevity in the world today, affecting up to 40% of the world’s population.^1^ With 80% of obese children predicted to grow into obese adults,^2^ the prevalence of individuals exposed to a lifetime of excess body weight is only expected to grow in the coming decades.

A wealth of recent literature has implicated obesity as a major risk factor for another of the world’s greatest health challenges—dementia.^3^ Numerous observational studies and meta-analyses have identified associations between exposure to obesity—particularly in the mid-life period—and an increased risk of Alzheimer’s disease and other dementias later in the lifespan. If confirmed to be causal, these findings would suggest that strategies to target obesity during mid-life may act to reduce dementia incidence in the following decades.

The exact mechanisms underpinning this increased risk remain unclear but are commonly thought to arise at least in part due to ongoing atrophy and other changes within the brain during the decades-long preclinical phase of dementia. In support of this, the presence of obesity in midlife has been repeatedly linked to lower brain volumes in both cortical and subcortical regions relative to intracranial volume (a proxy for maximal attained brain size),^4^ with these differences commonly taken to represent an ongoing atrophy of brain tissues in response to risk factor exposures.

An alternative explanation for differences in brain volumes measured in adulthood, however, is that they may represent preserved differentiation arising from developmental differences in early life, rather than an active atrophy process in later years. Indeed, many of the volumetric measures commonly used in adulthood are known to peak in early childhood and/or adolescence,^5^ and a number of emerging studies show that numerous early-life risk factors for dementia may be more closely related to adult brain health than contemporary risk factors, as recently summarized in the review by Walhovd *et al*.^6^

The vast majority of these studies to date, however, are observational in nature and are therefore liable to various well-established potential biases such as reverse causation or confounding. By using naturally occurring genetic variants as instrumental variables, Mendelian randomization (MR) attempts to overcome these limitations by exploiting the quasi-random assortment of genetic alleles at birth within a population. As these genetic alleles are typically fixed throughout the life course, they are less likely to be influenced by confounding factors or liable to reverse causation. Provided that a number of core assumptions are met, MR can therefore be used to attempt to estimate causal effects from observational data.^7^ Extending this approach further, the inclusion of genetic variants for multiple exposures in a single model using multivariable Mendelian randomization (MVMR) can further inform whether these effects potentially act through direct or indirect (i.e. mediated through other downstream exposures) pathways. Recently, it has been shown that childhood and adult adiposity can be separated using MVMR,^8^ allowing the development of a life course MVMR approach identifying the potential causal impact that adiposity at different stages of life exerts on a range of later-life disease outcomes.^9-14^

Using this approach in two large cohorts of adults and children with measures of both adiposity and volumetric neuroimaging phenotypes, this study aimed to investigate whether early-life differences in adiposity may explain adult brain volumetric differences commonly attributed to subclinical obesity-related disease processes.

## Materials and methods

### Study design

We first conducted a combination of observational, univariable and multivariable two-sample MR analyses in 37 501 middle-aged participants recruited to the UK Biobank (UKB) to assess the total and direct causal effects of adiposity in childhood (∼age 10) and adulthood (∼age 55) on numerous brain traits commonly used as subclinical markers of obesity-related neurodegenerative disease. Next, we used the same childhood genetic variants to construct a genetic risk score to test if these relationships between adiposity and brain traits were already evident at ∼10 years of age in 6996 children of European ancestry recruited to the Adolescent Brain Cognitive Development Study (ABCD). Details on the cohorts included in this study can be found in the publications by Sudlow *et al*.^15^ and Casey *et al*.^16^ Participants’ consent for this study was obtained according to the Declaration of Helsinki and had ethical approval from UKB covered under Application #71702 and from the ABCD covered under Data Access Request ID #13724.

### Genetic instruments for childhood and adult adiposity

Independent genome-wide association studies (GWASs) of childhood adiposity (derived using recall questionnaire data asking participants if they were ‘thinner’, ‘plumper’ or ‘about average’ when they were aged 10 years old) and adult adiposity [body mass index (BMI) converted into a categorical variable with cut-offs at <25 kg/m^2^, 25–31.7 kg/m^2^ and >31.7 kg/m^2^ to ensure comparability with childhood adiposity category] were separately conducted in the UKB based on conventional genome-wide corrections (i.e. *P* < 5 × 10^−8^). These analyses identified 295 and 557 independent variants in 453 169 participants for childhood and adult adiposity, respectively, have since been validated in three independent cohorts of young people using directly measured BMI^8,11,17^ and have been shown predominantly to represent the accumulation of fat mass rather than lean mass in the young.^12^ For the present study, original GWASs were repeated in the UKB after excluding participants with neuroimaging outcomes of interest, allowing the partitioning of the cohort into two independent samples for two-sample MR analyses and reducing the chance of overfitting in our data due to so-called ‘winner’s curse’. These new GWASs identified 266 and 470 variants for childhood and adult adiposity in 407 741 UKB participants following adjustments for age, sex, genotyping chip and (for childhood adiposity) month of birth. To account for genetic relatedness and geographical structure, a linear mixed model using BOLT-LMM software was employed to conduct each GWAS and genetic instruments were selected based on variants that met the criteria of *P* < 5 × 10^−8^ and with an R^2^ < 0.001 using a reference panel of *n* = 10 000 randomly selected unrelated European participants from UKB. For MVMR models, linkage disequilibrium clumping of both sets of genetic variants was performed (based on R^2^ < 0.001) prior to inclusion in the model to ensure independence of instruments, resulting in the inclusion of 229 and 435 variants in multivariable models for childhood and adult adiposity, respectively.

### Creation of a genetic risk score for childhood adiposity in the ABCD study

To maximize statistical power in secondary analyses of the smaller ABCD cohort, we used individual-level data to condense our instruments into a single, more powerful genetic risk score for analyses. These were restricted to those of European ancestry only, and Pedigree Reconstruction and Identification of a Maximum Unrelated Set (PRIMUS) analysis was used to calculate ancestry principal components (PCs). Individual-level genetic risk scores using 214 genetic variants for childhood adiposity were constructed using the –score function in Plink for each included participant that had corresponding phenotypic and covariate data available (*n* = 6609). Linear mixed-effects models using REML were employed to relate the childhood adiposity genetic risk score to brain volumetric measures while adjusting for age, sex and 10 PCs. A random intercept for family was included to account for relatedness between participants. In line with other inverse-variance weighted and observational techniques employed in the paper, the genetic risk score was categorized into ordinal categories comprising low (≤33rd percentile), mid (>33rd and <85th percentile) and high (≥85th percentile) groupings for analyses.

### Brain structural outcomes

In the UKB, structural MRI brain scans were available in 37 501 genotyped participants scanned as part of the imaging sub-study, described in more detail by Littlejohns *et al*.^18^ and in the Supplementary material. Primary outcomes selected were adult total brain volume; grey matter volume, thickness and surface area; white matter volume and hyperintensities; and hippocampus, amygdala and thalamus volumes at mean age 55. All volumes were indexed to intracranial volume (trait : intracranial volume) before inclusion in the main analyses, with the exception of cortical thickness and white matter hyperintensities.^19^ In ABCD, equivalent phenotypes were selected where possible from 6996 participants scanned at ages 9–10 years, described in more detail by Casey *et al*.^16^ and in the Supplementary material.

### Estimating total and direct effects of childhood and adult adiposity on adult brain outcomes

#### Observational estimates

Multivariable linear regression analyses adjusted for age, sex, highest education level achieved and Townsend deprivation index were used to cross-sectionally test associations between BMI and brain traits of interest in adulthood in the UKB. Three categories of BMI based on the same cut-offs as GWAS analyses (<25 kg/m^2^, 25–31.7 kg/m^2^, >31.7 kg/m^2^) were used to ensure comparability with genetic analyses as earlier described.

#### Total effects

Univariable two-sample MR was conducted using variant-exposure summary level data from UKB individuals without MRI imaging data (*n* = 407 741) and variant-outcome summary level data from those with imaging data (*n* = 37 501). Effects for childhood and adult adiposity were then independently estimated using the inverse-variance weighted method.

#### Direct effects

**Figure 1:**
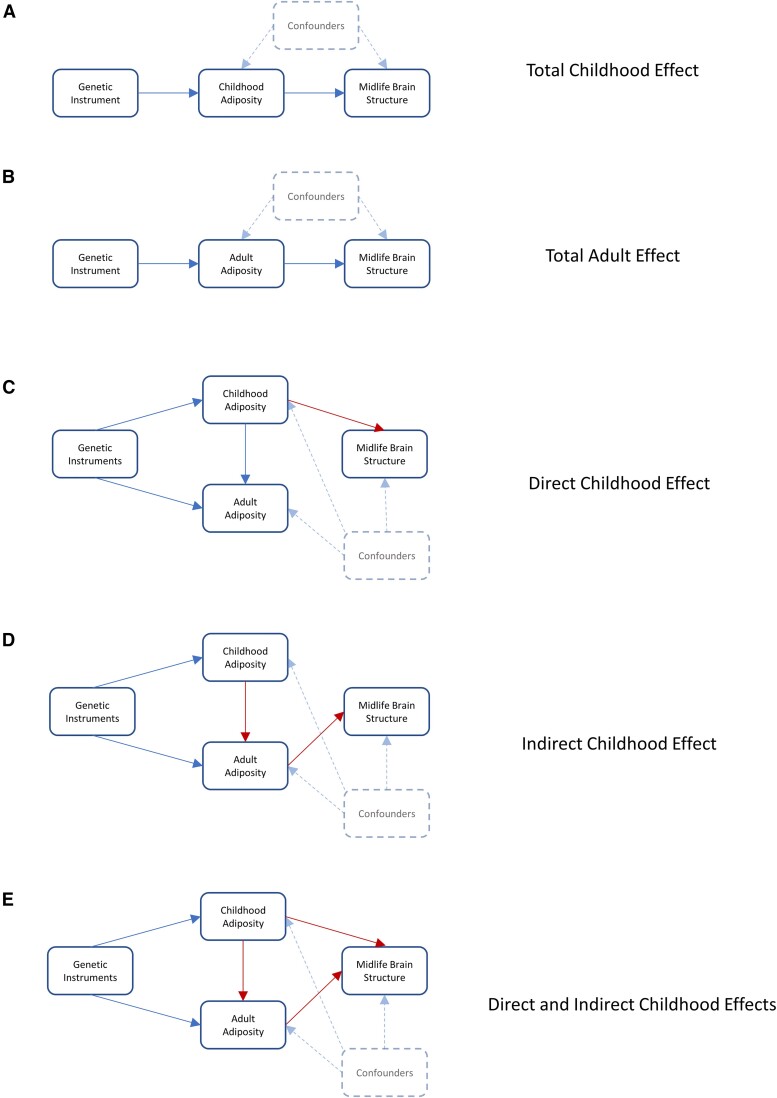
**Directed acyclic graphs illustrating potential paths by which childhood adiposity may impact adult brain traits**. Five directed acyclic graphs demonstrating potential ways in which childhood and adult adiposity may impact adult brain traits. **A** and **B** show the potential for univariable Mendelian randomization (MR) to assess the total effects of childhood and adult adiposity on adult brain traits. **C–E** show the ways in which multivariable MR may isolate the underlying causal effects responsible. In **C**, childhood adiposity exerts a direct effect on later brain traits while also separately influencing later-life adiposity. In **D**, childhood adiposity exerts an indirect effect on later brain traits mediated solely via its effect on later-life adiposity. In **E**, childhood adiposity exerts both direct effects on later brain traits and indirect effects mediated through the persistence of adiposity over time. The use of genetic variants as proxies for exposures minimizes confounding bias and allows causal effects to be estimated.

Multivariable two-sample MR was next employed to assess whether any observed associations between childhood adiposity and adult brain traits were likely due to direct effects of childhood adiposity itself or were instead mediated through the well-documented persistence of adiposity throughout the life-course.^2^ This technique uses the inclusion of genetic variants for both childhood and adult adiposity in the same MR model, thereby allowing the estimation of their independent genetically-predicted effects. Directed acyclic graphs demonstrating examples of potential pathways linking exposures to outcomes can be seen in Fig. 1.

### Estimating the total effect of childhood adiposity on childhood brain outcomes

#### Observational estimates

Multivariable linear regression analyses adjusted for age, sex, parents’ education, household income and model of MRI scanner were used to cross-sectionally test associations between BMI and brain traits of interest in children of European descent in ABCD. CDC growth charts were used to derive age- and sex-adjusted BMI exposures, which were then categorized into ≤33rd percentile, 33rd to 85th percentile and >85th percentile to broadly replicate adult stratification.

#### Total effects

A weighted genetic risk score for childhood adiposity was constructed in ABCD using 214 childhood adiposity genetic variants identified from UKB as detailed above. This genetic risk score was strongly associated with childhood BMI generated using CDC growth charts [beta (95% confidence interval, CI) = 0.32 (0.28, 0.36); *P* = 2 × 10^−58^], confirming its utility as a measure of childhood adiposity rather than simply growth.

### Sensitivity analyses

#### Violations of core Mendelian randomization assumptions

Violations of any of the three core assumptions of MR analyses (relevance, independence and exclusion restriction) may result in biased estimates and so must be tested using appropriate methods. Data pertaining to assumption 1 (relevance) have been reported previously^9^ and have shown F-statistics to be >10 in both univariable and multivariable models, suggesting that weak instrument bias is unlikely when using these instruments. To test for the potential presence of pleiotropy, which may violate assumption 2 (independence), we first assessed heterogeneity within our univariable models using Cochran’s Q statistic. Next, we compared all inverse-variance weighted analyses to models run using MR Egger, weighted median and weighted mode techniques. Furthermore, we generated funnel plots displaying the extent to which pleiotropy is balanced across instruments, with symmetry taken as evidence against a directional pleiotropic effect. Finally, we re-ran all analyses eliminating a single genetic variant in turn in a ‘leave-one-out’ analysis to test if any included variants were unduly affecting causal estimates.

#### Impact of indexing on brain outcomes

Brain structural phenotypes are commonly indexed/adjusted for some form of head size due to known associations between the two, but the way in which this normalization is performed can have implications for the interpretation of results. In addition to our primary outcomes in which the majority of our brain structures (exceptions white matter hyperintensity volume and cortical thickness^19^) were indexed to intracranial volume, we also re-ran all models either without any indexing (i.e. reporting absolute volumes) or when indexed to total brain volume so that findings could be compared.

#### Potential upstream effects of birthweight

Previous literature has also implicated prenatal growth and birthweight as important determinants of brain volume measured up to 70 years later.^20^ We, therefore, carried out additional MVMR analyses using genetic variants for birthweight as an exposure in our model alongside childhood adiposity to test for any potential effects of this upstream exposure. Genetic variants for birthweight were derived using the same analysis pipeline described above for childhood and adult adiposity but with birthweight kept as a continuous trait due to only being available in 261 932 UKB individuals. Birthweight was rank-based inverse normal transformed to ensure normality before adjustment for age, sex and genotyping chip as before.

### Statistical interpretation

*A priori,* we planned to base our interpretation of findings predominantly on effect estimates and their 95% CIs rather than arbitrarily assigning ‘significance/non-significance’ using a *P*-value cut-off. Exact *P*-values are presented throughout the manuscript, however, for full transparency. All univariable MR analyses were carried out using the ‘mrrobust’ package in Stata MP 18 (StataCorp LLC, Texas, USA), while multivariable MR analyses were conducted using the ‘TwoSampleMR’ package in R version 4.3.1 (R Core Team 2023, Vienna, Austria). Summary level data for variant-outcome associations in UKB were generated using PLINK2 while accounting for age, sex and population stratification using 10 genetic PCs.

## Results

Summary statistics for phenotypic exposures and outcomes used in the UKB and ABCD cohorts can be found in Supplementary Tables 1 and 2.

### Differences in childhood adiposity persist across the life course

In total, 77% of individuals in the top category for childhood adiposity were classed as being above normal weight at the time of their adult involvement in UKB, with around half of these qualifying as obese. In contrast, only 12% of individuals who reported being ‘thinner than average’ in childhood qualified as obese at the time of study. Adult height was similar across all groups reporting differences in adiposity during childhood (1.69 ± 0.09 m for all groups).

### Observational and univariable MR analyses link adult adiposity to brain traits in UKB

#### Observational

**Figure 2:**
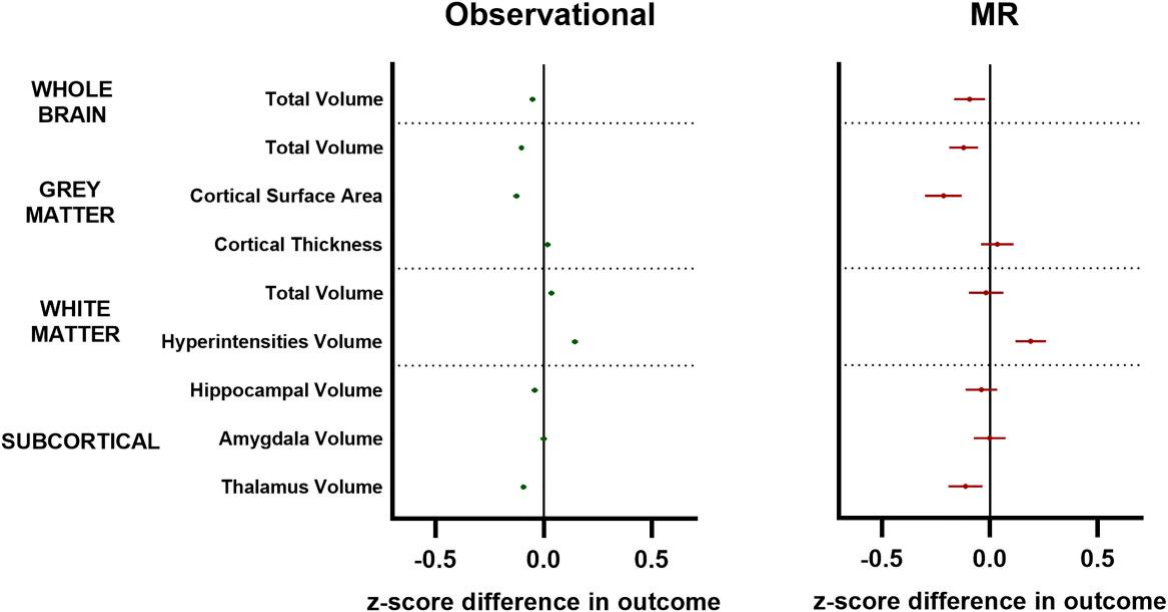
**Observational and univariable Mendelian randomization analyses of adult adiposity versus adult brain traits.** Forest plots showing cross-sectional observational (*left*) and Mendelian randomization (MR; *right*) analyses linking adult adiposity to adult brain traits in the UK Biobank. Symbols represent mean *z*-score difference in each outcome (95% confidence intervals) across categories of adiposity. Green symbols = observational data; red symbols = MR data. White matter hyperintensity volumes were log-transformed prior to analysis. All outcomes indexed to intracranial volume except for cortical thickness and white matter hyperintensities.

Multivariable linear regression models suggested a negative association between adiposity and numerous normalized brain volumes in adulthood [e.g. normalized grey matter volume beta (95% CI) = −0.05 (−0.06, −0.04); *P =* 2 × 10^−61^; Fig. 2, left].

#### Mendelian randomization

Estimates generated using two-sample univariable MR mirrored that of observational analyses, albeit with wider confidence intervals (Fig. 2, right).

### Multivariable MR analysis suggests childhood adiposity underlies many observed differences in UKB

**Figure 3:**
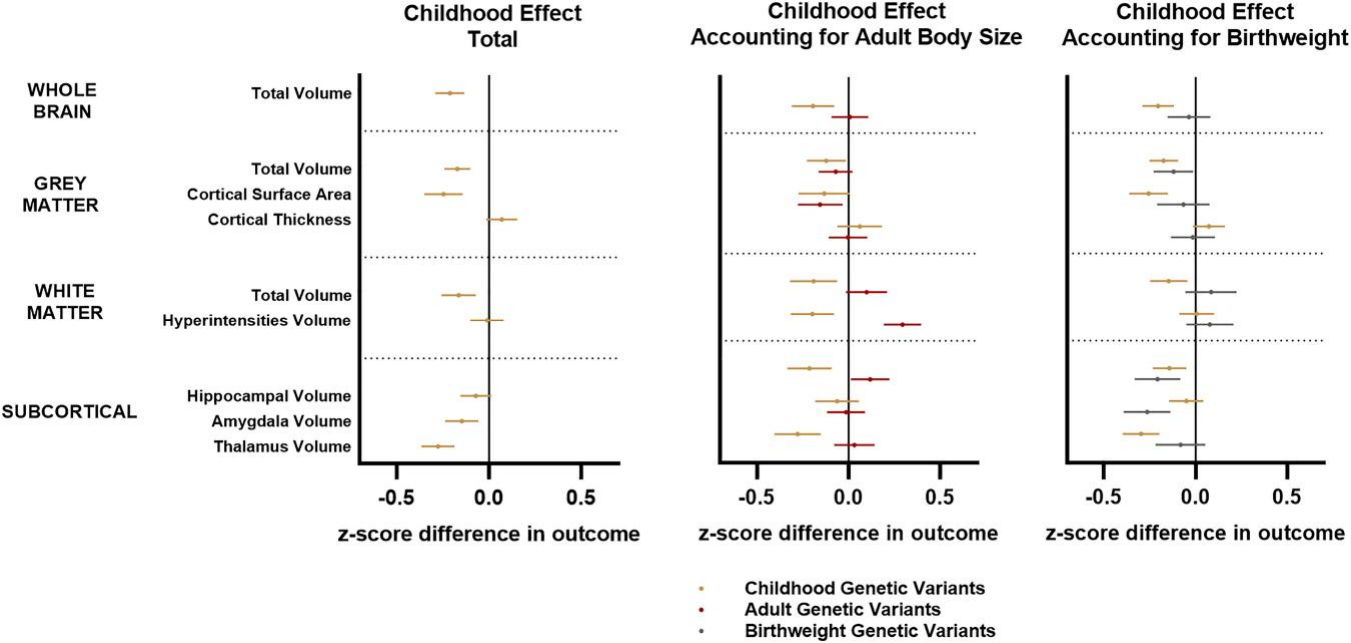
**Total and direct effects of childhood adiposity on adult brain traits after accounting for potential downstream (adult obesity) and upstream (birthweight) influential factors**. Forest plots showing univariable Mendelian randomization analyses assessing childhood total effects (*left*) and multivariable analyses assessing direct effects after accounting for adult adiposity (*middle*) and birthweight (*right*) on adult brain traits in the UK Biobank. Symbols represent mean *z*-score difference in each outcome (95% confidence intervals) across categories of adiposity. Gold symbols = childhood genetic variants; red symbols = adult genetic variants; grey symbols = birthweight genetic variants. White matter hyperintensity volumes were log-transformed prior to analysis. All outcomes indexed to intracranial volume except for cortical thickness and white matter hyperintensities.

Effect estimates for adult brain traits when using genetic variants for childhood adiposity were found to be of a similar or greater magnitude than adult variants for all outcomes measured [e.g. normalized grey matter volume beta (95% CI) = −0.17 (−0.25, −0.09); *P* = 9 × 10^−5^; Fig. 3, left]. The subsequent inclusion of both childhood and adult genetic variants in a multivariable MR model provided strong evidence for childhood adiposity as the primary causal factor underlying many differences in normalized brain volumes observed in adulthood, with prior total effects linking childhood adiposity to most brain traits remaining robust after accounting for adult adiposity (Fig. 3, middle and Supplementary Table 3). The strongest evidence for a direct effect of adult adiposity on any measured outcomes was in relation to increased levels of white matter hyperintensities [beta (95% CI) = 0.24 (0.13, 0.35); *P* = 3 × 10^−5^] and reductions in cortical surface area (Fig. 3, middle).

### Observational and genetic evidence for an effect of childhood adiposity on brain traits by age 10

#### Observational

**Figure 4:**
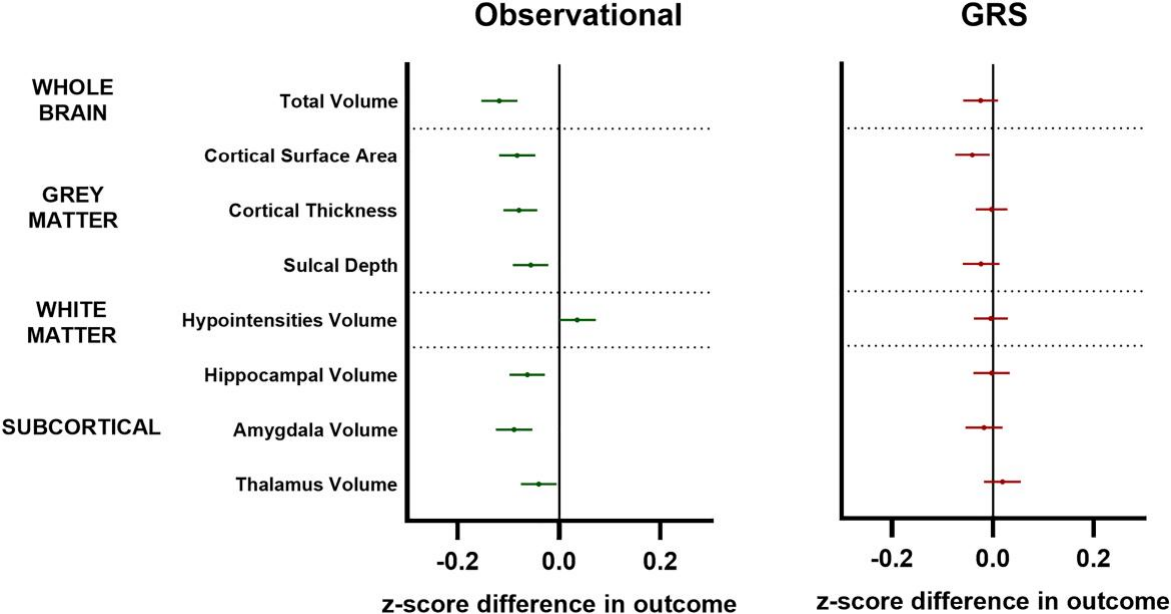
**Observational and genetic risk score analyses of childhood adiposity on childhood brain traits at age 10 years.** Forest plots showing cross-sectional observational (*left*) and genetic risk score (GRS; *right*) analyses linking childhood adiposity to childhood brain traits in the Adolescent Brain Cognitive Development Study. Symbols represent mean *z*-score difference in each outcome (95% confidence intervals) across categories of adiposity. Green symbols = observational data; red symbols = GRS data. White matter hypointensity volumes were log-transformed prior to analysis. All outcomes indexed to intracranial volume except for cortical thickness and white matter hypointensities.

Multivariable linear regression models suggested a negative association between adiposity and most measured brain traits that largely mirrored the pattern of total childhood effects observed in adulthood in the UKB (Fig. 4).

#### Genetic risk score

In line with observational analyses, we saw weak evidence of a negative relationship between our genetic risk score and whole brain volume, cortical surface area and sulcal depth [e.g. surface area beta (95% CI) = −0.04 (−0.08, −0.01); *P* = 0.02; Fig. 4].

### Sensitivity analyses

#### Testing MR assumptions

While all univariable analyses showed evidence of heterogeneity using Cochran’s Q statistic (Supplementary Tables 4, 6, 8, 10, 12 and 14), funnel plots for each outcome showed little-to-no evidence of directional pleiotropy in these differences, suggesting that the overall causal estimate for each outcome is unlikely to be biased (Supplementary Figs 3 and 4). Furthermore, MR Egger coefficients and intercepts largely matched those generated using inverse-variance weighted methods for all analyses (Supplementary Tables 3–15), suggesting that horizontal pleiotropy was unlikely to be a contributing factor. These findings were further supported by weighted median and mode estimates (Supplementary Tables 3–15) as well as leave-one-out analyses, which failed to identify any genetic variants exerting an undue influence on the results (Supplementary Figs 5 and 6).

#### Impact of normalizing brain traits

Higher adiposity in childhood was found to be linked to higher intracranial volumes and—to a lesser extent—cortical volumes in UKB, suggesting a potential mismatching of head and brain growth in those who were large as children (Supplementary Figs 1 and 2 and Supplementary Tables 16 and 17). These findings were confirmed observationally in ABCD, with the largest children at 10 years old found to have modestly larger cortical volumes but even larger intracranial volumes.

#### Upstream impact of birthweight

Univariable MR showed evidence of a causal effect of birthweight on multiple volumetric markers of adult brain structure (Fig. 3, right and Supplementary Table 18) as has been suggested previously. However, the inclusion of these birthweight genetic variants into an MVMR model alongside childhood variants had a minimal effect on most childhood adiposity estimates, suggesting that our observed effects in childhood were not driven purely by a continuation of prior differences in body size at birth.

## Discussion

Our study suggests that numerous differences in volumetric brain traits commonly used as markers of ongoing subclinical disease from midlife onwards may, in fact, be the legacy effects of childhood adiposity and weight gain. Importantly, our sensitivity analyses suggest that these differences may occur due to the development of a larger head-to-brain size ratio in those with excess weight gain in childhood, rather than demonstrating the presence of atrophy and a smaller brain size *per se* in adulthood. Our findings do, however, provide some support for a causal role for adult adiposity in the ongoing development of white matter hyperintensities (a well-established marker of subclinical damage of presumed vascular origin), as well as evidence for ongoing reductions in grey matter cortical surface area.

Volumetric measures in cross-sectional neuroimaging cohorts that seek to link risk factors such as BMI to biomarkers of brain health are traditionally corrected for some measure of head size prior to analysis due to the well-established scaling between these traits.^4,21^ With intracranial volume commonly regarded as a proxy for maximal-attained brain size in childhood,^22^ any reductions in brain volumes relative to head size are thereby interpreted to represent evidence of a contemporary ageing or disease-related atrophy process that may putatively be linked to the risk factor of interest. In the current study, our initial analyses suggested some evidence of this phenomenon in the UKB in relation to adult obesity, with both observational and MR analyses showing smaller volumes in several neuroimaging traits relative to intracranial volume, particularly with regard to cortical grey matter volume and surface area. The majority of observed effects largely attenuated towards null after the inclusion of genetic variants for childhood adiposity in multivariable MR models, however, suggesting that excess body weight in childhood may instead directly influence a number of adult brain outcomes through the long-term preserved differentiation of imaging traits which persist into later life. In light of the well-established tracking of body size from conception onwards (whereby large children are more likely to have already been large babies^23^), we also ran additional multivariable MR models incorporating birthweight genetic variants alongside childhood adiposity in order to test whether observed effects were simply a legacy of upstream effects. Although these were also found to be causally linked to several volumetric measures, their inclusion had little effect on the strength of childhood estimates, suggesting that weight gain both *in utero* and during the first decade of life may exert long-term and independent effects on numerous commonly used volumetric traits. These findings were strengthened further in a secondary analysis of the childhood ABCD cohort, where observational and—to a lesser extent—genetic risk score analyses demonstrated a similar pattern across all measured outcomes that was already evident by the age of 10 years.

Our finding of an association between adiposity in the first decade of life and reduced normalized brain volumes in adulthood was perhaps surprising given the well-documented positive relationship linking early-life growth to head and brain dimensions,^24^ plus the known genetic overlap between both birthweight and BMI with larger brain volumes.^25^ We, therefore, re-ran all analyses without adjustment for intracranial volumes to assess the impact that our genetic variants for childhood and adult adiposity had on overall head and brain size. We found that individuals who were genetically predicted to have been larger either at birth or in childhood were found to have larger heads alongside modestly greater volumes in cortical—but not subcortical—regions compared to their peers. These findings once again mirrored those seen at age 10 in observational analyses of the childhood ABCD study and also support recent observational data from the Lothian Birth Cohort 1936, in which differences in brain volumes at age 73 were suggested to occur due to the presence of larger heads in those who were born heavier seven decades earlier, rather than representing evidence of ongoing atrophy in those who were born lighter.^20^ Our genetic data provide causal support for these observations, with univariate MR analyses using genetic variants for birthweight demonstrating strong positive associations with brain volumes that largely disappeared when assessing as a proportion of head size. We further expand upon these findings, however, by demonstrating for the first time that further excess weight gain in the next 10 years of childhood may result in the development of a lower maximal attained brain-to-head size ratio, an observation which may inadvertently be interpreted as evidence of atrophy in later-life studies where brain volumes are routinely normalized for intracranial volume.

Why weight gain in childhood would drive this phenomenon is speculative but is supported by numerous observations in the literature. For example, in a study of >2000 children followed across the early years of life, a consistent relationship between head circumference and body mass has been demonstrated, resulting in an almost constant ratio between head circumference cubed/body mass which is maintained across the first 18 months of life.^26^ Moving beyond the early years, it is well-documented that increases in head size continue until ∼age 18 years or even beyond, despite brain volume peaking many years earlier at around 11 years.^27^ As a result, the ratio of brain-to-CSF volume within the cranium has been shown to decrease steadily over the teenage years and into adulthood,^24,28^ a finding which may explain the mismatch between head size and brain volumes seen in our adult cohort. This timing of events may also explain the weak effect sizes seen in the genetic risk score analysis of our younger ABCD cohort compared to that seen in the UKB, as our childhood measures at age 10 may have been slightly too early to detect changes known to occur in adolescence. The exact changes underlying this head-to-brain size ratio in adolescence remain to be determined but may arise from (i) mechanisms such as synaptic pruning that are known to occur during adolescent development; (ii) accelerations in cranial growth that have been documented in obese children at this age^29^; or (iii) differences in non-tissue spaces within the skull such as the ventricles.

Whether such weight gain in childhood has any long-term clinical implications for the development of conditions such as dementia also remains to be determined. Indeed, despite our primary analyses suggesting the presence of a persistently reduced brain : intracranial volume ratio in those who reported higher adiposity as children, sensitivity analyses in both the UKB and ABCD cohorts consistently demonstrated that total brain volumes in these individuals tended to be similar or larger overall—raising the question as to whether accelerated weight gain in the early years may, in fact, contribute to an increased initial brain reserve as has been previously suggested.^20^ It should be noted, however, that these volumetric increases were confined to the cortex, with no apparent accompanying increase in a number of subcortical structures with important contributions to cognitive function (e.g. the hippocampus^30^ and thalamus^31^). In adulthood, our analyses consistently highlighted a decreased grey matter surface area and increased white matter hyperintensity burden in obese adults that persisted regardless of normalization and even after accounting for childhood adiposity. These findings support ongoing obesity-related changes within these areas and backup claims of midlife as a sensitive period for the effects of obesity on certain forms of cerebral damage.

### Limitations

Our study has a number of strengths and limitations. First, due to a lack of childhood measurements in UKB, genetic variants for childhood adiposity were based on questionnaire-based recall of an individual’s approximate body size at age 10—a technique which is likely imprecise and subject to recall and other biases. Multiple follow-up studies since publication of the original GWAS, however, have validated the use of these variants as strong predictors of BMI in early life, capable of separating effects from adulthood BMI.^11,12^ Moreover, childhood effects have been shown to be driven more by a gain of fat tissue rather than lean mass (e.g. adiposity rather than growth)^12^ during childhood and adolescence, highlighting that these variants capture effects of adiposity in childhood rather than overall body size. We further confirmed these findings in our current ABCD cohort, with our genetic risk score showing extremely strong relationships with BMI *z*-scores even after accounting for childhood growth (Supplementary material), suggesting that our instrument is predominantly capturing weight gain prior to age 10 years. Second, numerous methods exist for normalizing brain volumes to body dimensions, each with their own advantages, drawbacks and biases.^32^ Here, we chose to use the proportion method (i.e. trait : intracranial volume) rather than the residual method. This was because our aim was to test whether any observed volumetric differences were due to ongoing atrophy of brain tissues relative to ‘maximal attained brain size’ (i.e. intracranial volume) rather than trying to remove any potential confounding effect of head size itself.^33^ Third, MR using fetal genetic variants for birthweight only directly addresses fetal contributions to outcomes and does not capture the differential effects of maternal influences (influenced by maternal genetics), which may also play a role.^34^ Finally, while results from our observational analyses at age 10 closely mirrored effects seen in adulthood in those who were genetically predicted to have been larger as children, follow-up analyses using our childhood genetic risk score were less conclusive. This may be due to the reduced power offered by the smaller ABCD dataset (∼7000 versus ∼38 000 for UKB) or the age at which outcomes were measured (as discussed earlier) but also feasibly represents a true finding where differences seen at this age are not necessarily causally linked to adiposity *per se.* However—regardless of the underlying mechanisms—our observational data demonstrate that larger children already appear to have smaller brain volumes relative to their head size at age 10, despite having marginally larger brains overall. These findings mirror that seen in our UKB analyses many decades later, supporting this phenomenon as one which is established and then persists from the early years, as opposed to an ongoing atrophy process in later life.

## Conclusions

Differences in brain traits commonly used as markers of subclinical disease in adulthood may in fact be legacy effects of childhood growth and developmental processes. Caution should be warranted when using cross-sectional neuroimaging measures to represent ongoing disease processes.

## Supplementary Material

awae198_Supplementary_Data

## Data Availability

All data used in this publication are open access and available to *bona fide* researchers through well-documented processes detailed at https://www.ukbiobank.ac.uk/ and https://abcdstudy.org/ for UKB and ABCD, respectively. The ABCD data repository grows and changes over time. The ABCD data used in this report came from DOI: 10.15154/g3ht-mp98. DOIs can be found at https://dx.doi.org/. The statistical code for all analyses in this paper can be found in an open-access GitHub repository located at https://github.com/scottchiesa/lifecourse-MR-brain.
