# Supplementary material for: Childhood adiposity underlies numerous adult brain traits commonly attributed to midlife obesity
Source: Brain. 2024 Jun 18;148(1):133–42. doi: 10.1093/brain/awae198 (PMC11706278; doi:10.1093/brain/awae198)

Childhood Adiposity Underlies Numerous Adult Brain Traits Commonly Attributed to Midlife Obesity: A Lifecourse Mendelian Randomization Study

Scott T. Chiesa PhD[1], Lydia Rader MSc[2], Victoria Garfield PhD[1], Isabelle Foote PhD[2], Sana Suri DPhil[3,4], George Davey Smith PhD[5], Alun D. Hughes PhD[1], and Tom G. Richardson PhD[5]

TABLE OF CONTENTS

SUPPLEMENTAL METHODS 4

Brain Structural Outcomes in UKB and ABCD 4

SUPPLEMENTAL TABLES 6

Supplemental Table 1: Descriptive Statistics for Exposures and Outcomes of Interest in UKB 6

Supplemental Table 2: Descriptive Statistics for Exposures and Outcomes of Interest in ABCD 7

Supplemental Table 3: MVMR Results Demonstrating Effects of Childhood and Adult Adiposity on Midlife Brain Traits 8

Supplemental Table 4: MR Egger Intercepts and Cochran's Q Statistics for Total Effect of Childhood Adiposity on Midlife Brain Traits 9

Supplemental Table 5: Beta Estimates from Sensitivity Analyses for Total Effect of Childhood Adiposity on Midlife Brain Traits 10

Supplemental Table 6: MR Egger Intercepts and Cochran's Q Statistics for Total Effect of Adult Adiposity on Midlife Brain Traits 11

Supplemental Table 7: Beta Estimates from Sensitivity Analyses for Total Effect of Adult Adiposity on Midlife Brain Traits 12

Supplemental Table 8: MR Egger Intercepts and Cochran's Q Statistics for Total Effect of Childhood Adiposity on Midlife Brain Traits Without Any Form of Head Size Adjustment 13

Supplemental Table 9: Sensitivity Analyses for Total Effect of Childhood Adiposity on Midlife Brain Traits Without Any Form of Head Size Adjustment 14

Supplemental Table 10: MR Egger Intercepts and Cochran's Q Statistics for Total Effect of Adult Adiposity on Midlife Brain Traits Without Any Form of Head Size Adjustment 15

Supplemental Table 11: Sensitivity Analyses for Total Effect of Adult Adiposity on Midlife Brain Traits Without Any Form of Head Size Adjustment 16

Supplemental Table 12: MR Egger Intercepts and Cochran's Q Statistics for Total Effect of Childhood Adiposity on Midlife Brain Traits when Outcomes Adjusted for Total Brain Volume Instead of Intracranial Volume 17

Supplemental Table 13: Sensitivity Analyses for Total Effect of Childhood Adiposity on Midlife Brain Traits when Outcomes Adjusted for Total Brain Volume Instead of Intracranial Volume 18

Supplemental Table 14: MR Egger Intercepts and Cochran's Q Statistics for Total Effect of Adult Adiposity on Midlife Brain Traits when Outcomes Adjusted for Total Brain Volume Instead of Intracranial Volume 19

Supplemental Table 15: Sensitivity Analyses for Total Effect of Adult Adiposity on Midlife Brain Traits when Outcomes Adjusted for Total Brain Volume Instead of Intracranial Volume 20

Supplemental Table 16: MVMR Results Demonstrating Effects of Childhood and Adult Adiposity on Midlife Brain Traits Without Any Form of Head Size Adjustment 21

Supplemental Table 17: MVMR Results Demonstrating Effects of Childhood and Adult Adiposity on Midlife Brain Traits when Outcomes Adjusted for Total Brain Volume Instead of Intracranial Volume . 22

Supplemental Table 18: MVMR Results Demonstrating Effects of Birthweight and Childhood Adiposity on Midlife Brain Traits 23

SUPPLEMENTAL FIGURES 24

Supplemental Figure 1: Forest Plots for Total and Direct Effects of Childhood and Adult Adiposity on Midlife Brain Traits Without Adjustment for Intracranial Volume 24

Supplemental Figure 2: Forest Plots for Total Effects of Childhood and Adult Adiposity on Midlife Brain Traits when Outcomes Adjusted for Total Brain Volume 25

Supplemental Figure 3: Funnel Plots demonstrating balance of pleiotropic effects for each brain trait in main analysis across all childhood instruments 26

Supplemental Figure 4: Funnel Plots demonstrating balance of pleiotropic effects for each brain trait in main analysis across all adult instruments 27

Supplemental Figure 5: Leave-one-out analyses to test for undue influence of single genetic variants which may potentially impact main analysis of childhood total effects 28

Supplemental Figure 6: Leave-one-out analyses to test for undue influence of single genetic variants which may potentially impact main analysis of adult total effects 31

SUPPLEMENTAL METHODS

Brain Structural Outcomes in UKB and ABCD

The following image-derived phenotypes were selected from UKB to be used as outcomes in the current study: total brain volume (UKB ID# n_25010_2_0; mm^3); grey matter volume (UKB ID# n_25006_2_0; mm^3), surface area (mean of UKB ID#s n_26721_2_0 and n_26822_2_0; mm^2), and cortical thickness (mean of UKB ID#s n_26755_2_0 and n_26856_2_0; mm); white matter volume (UKB ID# n_25008_2_0; mm^3) and hyperintensity volume (UKB ID# n_25781_2_0; mm^3); and subcortical volumes in the amygdala (mean of UKB ID#s n_25021_2_0 and n_25022_2_0; mm^3), hippocampus (mean of UKB ID#s n_25019_2_0 and n_25020_2_0, mm^3), and thalamus (mean of n_25011_2_0 and n_25012_2_0; mm^3). White matter hyperintensities were positively skewed and so were natural log-transformed prior to inclusion in models. Intracranial volume (UKB ID# n_26521_2_0; mm^3) was also measured to account for maximal attained brain size in subsequent analyses, with normalized outcomes reported as outcome/ICV. In ABCD, equivalent phenotypes were chosen from baseline measured collected at age 9-10 years to match those measured in UKB as closely as possible, resulting in the following phenotypes of interest: total brain volume (ABCD ID# smri_vol_scs_wholeb; mm^3); surface area (ABCD ID# smri_area_cdk_total; mm^2); cortical thickness (ABCD ID# smri_thick_cdk_mean; mm) and sulcal depth (ABCD ID# smri_sulc_cdk_mean; mm); white matter hypointensity volume (ABCD ID# smri_vol_scs_wmhint; mm^3); and subcortical volumes in the amygdala (mean of ABCD ID#s smri_vol_scs_amygdalalh and smri_vol_scs_amygdalarh; mm^3), hippocampus (mean of ABCD ID#s smri_vol_scs_hpuslh and smri_vol_scs_hpusrh; mm^3), and thalamus (mean of ABCD ID#s smri_vol_scs_tplh and smri_vol_scs_tprh; mm^3). White matter hypointensities were positively skewed and so were natural log-transformed prior to inclusion in models. Intracranial volume (ABCD ID# smri_vol_scs_intracranialv; mm^3) was also measured to account for maximal attained brain size in subsequent analyses, with normalized volumes once again

reported as outcome/ICV. All outcomes were converted to z-scores prior to analysis to allow direct comparisons of effect sizes across different traits.

SUPPLEMENTAL TABLES

Supplemental Table 1: Descriptive Statistics for Exposures and Outcomes of Interest in UKB

**Phenotype**	**Mean ± SD or (%)**
Age (years)	54.9 ± 7.5
Sex (% female)	47
BMI (kg/m^2)	26.6 ± 4.2
Total Brain Volume (mm^3)	1156706 ± 111554
Grey Matter Volume (mm^3)	612847 ± 55945
Cortical Surface Area (mm^2)	168650 ± 15572
Cortical Thickness (mm)	2.7 ± 0.1
White Matter Volume (mm^3)	543858 ± 61565
White Matter Hypointensities Volume (mm^3)	5172 ± 6781
Hippocampus Volume (mm^3)	3818 ± 446
Amygdala Volume (mm^3)	1243 ± 218
Thalamus Volume (mm^3)	7626 ± 746
Intracranial Volume (mm^3)	1546236 ± 153509

Supplemental Table 2: Descriptive Statistics for Exposures and Outcomes of Interest in ABCD

Phenotype	Mean ± SD or (%)
Age (years)	9.9 ± 0.6
Sex (% female)	47
BMI (kg/m^2)	18.2 ± 3.6
BMI-CDC (z-score)	0.3 ± 1.1
Total Brain Volume (mm^3)	1244904 ± 847476
Cortical Surface Area (mm^2)	192653 ± 17672
Cortical Thickness (mm)	2.7 ± 0.1
Sulcal Depth (mm)	-0.01 ± 0.01
White Matter Hypointensities Volume (mm^3)	865 ± 632
Hippocampus Volume (mm^3)	4148 ± 386
Amygdala Volume (mm^3)	1805 ± 214
Thalamus Volume (mm^3)	7643 ± 706
Intracranial Volume (mm^3)	1513770 ± 140504

Supplemental Table 3: MVMR Results Demonstrating Effects of Childhood and Adult Adiposity on Midlife Brain Traits

Outcome	Age	Beta	SE	p
Total Volume	Childhood	-0.19	0.06	0.001
Total Volume	Adult	0.01	0.05	0.9
Grey Matter Volume	Childhood	-0.12	0.05	0.03
Grey Matter Volume	Adult	-0.07	0.05	0.1
Cortical Surface Area	Childhood	-0.13	0.07	0.06
Cortical Surface Area	Adult	-0.15	0.06	0.013
Cortical Thickness	Childhood	0.06	0.06	0.3
Cortical Thickness	Adult	0.00	0.05	1.0
White Matter Volume	Childhood	-0.19	0.07	0.004
White Matter Volume	Adult	0.10	0.06	0.09
White Matter Hyperintensities	Childhood	-0.20	0.06	9.54E-04
White Matter Hyperintensities	Adult	0.29	0.05	1.36E-08
Amygdala	Childhood	-0.06	0.06	0.3
Amygdala	Adult	-0.01	0.05	0.8
Hippocampus	Childhood	-0.21	0.06	6.02E-04
Hippocampus	Adult	0.12	0.05	0.027
Thalamus	Childhood	-0.28	0.06	1.78E-05
Thalamus	Adult	0.03	0.06	0.6

Supplemental Table 4: MR Egger Intercepts and Cochran's Q Statistics for Total Effect of Childhood Adiposity on Midlife Brain Traits

Outcome	MR Egger Intercept	p	Cochran's Q	p
Total Brain Volume	0.001	0.4	391	<0.001
Grey Matter Volume	0.001	0.3	366	<0.001
Cortical Surface Area	0.000	0.8	538	<0.001
Cortical Thickness	0.001	0.6	426	<0.001
White Matter Volume	0.001	0.6	440	<0.001
White Matter Hyperintensities	0.001	0.5	514	<0.001
Hippocampus	-0.001	0.7	431	<0.001
Amygdala	0.000	0.9	332	0.003
Thalamus	0.000	0.9	452	<0.001

Supplemental Table 5: Beta Estimates from Sensitivity Analyses for Total Effect of Childhood Adiposity on Midlife Brain Traits

Outcome	No. SNPs	Method	Beta	SE	p
Total Brain Volume	266	Inverse variance weighted	-0.21	0.04	1.14E-07
Total Brain Volume	266	MR Egger	-0.29	0.09	0.001
Total Brain Volume	266	Weighted median	-0.22	0.06	1.01E-04
Total Brain Volume	266	Weighted mode	-0.29	0.09	8.06E-04
Grey Matter Volume	266	Inverse variance weighted	-0.17	0.04	2.04E-06
Grey Matter Volume	266	MR Egger	-0.24	0.08	0.003
Grey Matter Volume	266	Weighted median	-0.20	0.06	2.58E-04
Grey Matter Volume	266	Weighted mode	-0.25	0.08	2.21E-03
Cortical Surface Area	266	Inverse variance weighted	-0.25	0.05	3.10E-06
Cortical Surface Area	266	MR Egger	-0.21	0.12	0.07
Cortical Surface Area	266	Weighted median	-0.27	0.07	8.83E-05
Cortical Surface Area	266	Weighted mode	-0.30	0.13	0.02
Cortical Thickness	266	Inverse variance weighted	0.07	0.04	0.1
Cortical Thickness	266	MR Egger	0.02	0.10	0.8
Cortical Thickness	266	Weighted median	0.06	0.07	0.3
Cortical Thickness	266	Weighted mode	0.01	0.10	0.9
White Matter Volume	266	Inverse variance weighted	-0.16	0.05	4.95E-04
White Matter Volume	266	MR Egger	-0.21	0.11	0.05
White Matter Volume	266	Weighted median	-0.21	0.07	0.002
White Matter Volume	266	Weighted mode	-0.19	0.09	0.04
White Matter Hyperintensities	266	Inverse variance weighted	-0.01	0.05	0.8
White Matter Hyperintensities	266	MR Egger	-0.08	0.10	0.4
White Matter Hyperintensities	266	Weighted median	-0.01	0.06	0.9
White Matter Hyperintensities	266	Weighted mode	0.00	0.07	1.0
Hippocampus	266	Inverse variance weighted	-0.15	0.05	0.001
Hippocampus	266	MR Egger	-0.11	0.10	0.3
Hippocampus	266	Weighted median	-0.15	0.07	0.02
Hippocampus	266	Weighted mode	-0.11	0.09	0.2
Amygdala	266	Inverse variance weighted	-0.07	0.04	0.1
Amygdala	266	MR Egger	-0.06	0.10	0.5
Amygdala	266	Weighted median	-0.10	0.07	0.1
Amygdala	266	Weighted mode	-0.14	0.10	0.1
Thalamus	266	Inverse variance weighted	-0.28	0.05	1.17E-09
Thalamus	266	MR Egger	-0.29	0.10	0.005
Thalamus	266	Weighted median	-0.20	0.06	0.001
Thalamus	266	Weighted mode	-0.19	0.09	0.03

Supplemental Table 6: MR Egger Intercepts and Cochran's Q Statistics for Total Effect of Adult Adiposity on Midlife Brain Traits

Outcome	MR Egger Intercept	p	Cochran's Q	p
Total Brain Volume	0.002	0.2	811	<0.001
Grey Matter Volume	0.001	0.5	806	<0.001
Cortical Surface Area	0.001	0.8	900	<0.001
Cortical Thickness	0.000	0.8	856	<0.001
White Matter Volume	0.002	0.2	822	<0.001
White Matter Hyperintensities	0.002	0.1	775	<0.001
Hippocampus	0.002	0.1	719	<0.001
Amygdala	0.000	0.9	622	<0.001
Thalamus	0.003	0.07	874	<0.001

Supplemental Table 7: Beta Estimates from Sensitivity Analyses for Total Effect of Adult Adiposity on Midlife Brain Traits

Outcome	No. SNPs	Method	Beta	SE	p
Total Brain Volume	470	Inverse variance weighted	-0.09	0.04	9.91E-03
Total Brain Volume	470	MR Egger	-0.23	0.11	0.03
Total Brain Volume	470	Weighted median	-0.14	0.05	0.005
Total Brain Volume	470	Weighted mode	-0.21	0.13	0.1
Grey Matter Volume	470	Inverse variance weighted	-0.12	0.03	3.32E-04
Grey Matter Volume	470	MR Egger	-0.18	0.10	0.06
Grey Matter Volume	470	Weighted median	-0.15	0.05	0.002
Grey Matter Volume	470	Weighted mode	-0.21	0.10	0.03
Cortical Surface Area	470	Inverse variance weighted	-0.21	0.04	7.31E-07
Cortical Surface Area	470	MR Egger	-0.25	0.13	0.05
Cortical Surface Area	470	Weighted median	-0.18	0.06	9.29E-04
Cortical Surface Area	470	Weighted mode	-0.17	0.14	0.2
Cortical Thickness	470	Inverse variance weighted	0.03	0.04	0.4
Cortical Thickness	470	MR Egger	0.01	0.11	0.92
Cortical Thickness	470	Weighted median	0.07	0.05	0.2
Cortical Thickness	470	Weighted mode	0.13	0.12	0.
White Matter Volume	470	Inverse variance weighted	-0.02	0.04	0.7
White Matter Volume	470	MR Egger	-0.18	0.12	0.1
White Matter Volume	470	Weighted median	-0.02	0.05	0.7
White Matter Volume	470	Weighted mode	-0.01	0.15	0.9
White Matter Hyperintensities	470	Inverse variance weighted	0.19	0.04	1.82E-07
White Matter Hyperintensities	470	MR Egger	0.04	0.11	0.7
White Matter Hyperintensities	470	Weighted median	0.14	0.05	0.005
White Matter Hyperintensities	470	Weighted mode	0.11	0.10	0.2
Hippocampus	470	Inverse variance weighted	0.00	0.04	1.0
Hippocampus	470	MR Egger	-0.17	0.11	0.1
Hippocampus	470	Weighted median	-0.08	0.06	0.2
Hippocampus	470	Weighted mode	-0.12	0.10	0.2
Amygdala	470	Inverse variance weighted	-0.04	0.04	0.3
Amygdala	470	MR Egger	-0.03	0.11	0.8
Amygdala	470	Weighted median	-0.11	0.06	0.04
Amygdala	470	Weighted mode	-0.22	0.11	0.06
Thalamus	470	Inverse variance weighted	-0.11	0.04	4.70E-03
Thalamus	470	MR Egger	-0.31	0.12	0.007
Thalamus	470	Weighted median	-0.14	0.06	0.01
Thalamus	470	Weighted mode	-0.15	0.10	0.1

Supplemental Table 8: MR Egger Intercepts and Cochran's Q Statistics for Total Effect of Childhood Adiposity on Midlife Brain Traits Without Any Form of Head Size Adjustment

Outcome	MR Egger Intercept	p	Cochran's Q	p
Intracranial Volume	0.000	0.7	584	<0.001
Total Brain Volume	0.000	0.9	538	<0.001
Grey Matter Volume	0.000	0.8	536	<0.001
Cortical Surface Area	-0.001	0.7	566	<0.001
White Matter Volume	0.000	1.0	513	<0.001
Hippocampus	-0.001	0.5	404	<0.001
Amygdala	0.000	0.8	322	<0.001
Thalamus	0.000	0.9	481	<0.001

Supplemental Table 9: Sensitivity Analyses for Total Effect of Childhood Adiposity on Midlife Brain Traits Without Any Form of Head Size Adjustment

Outcome	No. SNPs	Method	Beta	SE	p
Intracranial Volume	266	Inverse variance weighted	0.14	0.05	0.002
Intracranial Volume	266	MR Egger	0.17	0.10	0.1
Intracranial Volume	266	Weighted median	0.11	0.06	0.05
Intracranial Volume	266	Weighted mode	0.10	0.09	0.3
Total Brain Volume	266	Inverse variance weighted	0.05	0.04	0.2
Total Brain Volume	266	MR Egger	0.03	0.10	0.7
Total Brain Volume	266	Weighted median	-0.05	0.06	0.4
Total Brain Volume	266	Weighted mode	-0.05	0.08	0.5
Grey Matter Volume	266	Inverse variance weighted	0.05	0.04	0.3
Grey Matter Volume	266	MR Egger	0.02	0.10	0.8
Grey Matter Volume	266	Weighted median	-0.05	0.06	0.4
Grey Matter Volume	266	Weighted mode	-0.10	0.08	0.2
Cortical Surface Area	266	Inverse variance weighted	0.02	0.04	0.7
Cortical Surface Area	266	MR Egger	0.05	0.10	0.6
Cortical Surface Area	266	Weighted median	0.02	0.06	0.8
Cortical Surface Area	266	Weighted mode	-0.01	0.08	0.9
White Matter Volume	266	Inverse variance weighted	0.07	0.04	0.1
White Matter Volume	266	MR Egger	0.02	0.10	0.8
White Matter Volume	266	Weighted median	0.06	0.07	0.3
White Matter Volume	266	Weighted mode	0.01	0.10	0.9
Hippocampus	266	Inverse variance weighted	0.05	0.04	0.2
Hippocampus	266	MR Egger	0.05	0.10	0.6
Hippocampus	266	Weighted median	0.04	0.06	0.6
Hippocampus	266	Weighted mode	0.00	0.08	1.0
Amygdala	266	Inverse variance weighted	-0.01	0.05	0.8
Amygdala	266	MR Egger	-0.08	0.10	0.4
Amygdala	266	Weighted median	-0.01	0.06	0.9
Amygdala	266	Weighted mode	0.00	0.07	1.0
Thalamus	266	Inverse variance weighted	-0.03	0.04	0.5
Thalamus	266	MR Egger	0.03	0.10	0.8
Thalamus	266	Weighted median	-0.11	0.06	0.08
Thalamus	266	Weighted mode	-0.08	0.09	0.3

Supplemental Table 10: MR Egger Intercepts and Cochran's Q Statistics for Total Effect of Adult Adiposity on Midlife Brain Traits Without Any Form of Head Size Adjustment

Outcome	MR Egger Intercept	p	Cochran's Q	p
Intracranial Volume	-0.001	0.4	947	<0.001
Total Brain Volume	0.000	0.8	943	<0.001
Grey Matter Volume	-0.001	0.7	905	<0.001
Cortical Surface Area	-0.001	0.5	955	<0.001
White Matter Volume	0.000	1.0	940	<0.001
Hippocampus	0.001	0.4	782	<0.001
Amygdala	-0.001	0.6	681	<0.001
Thalamus	0.001	0.4	920	<0.001

Supplemental Table 11: Sensitivity Analyses for Total Effect of Adult Adiposity on Midlife Brain Traits Without Any Form of Head Size Adjustment

Outcome	No. SNPs	Method	Beta	SE	p
Intracranial Volume	470	Inverse variance weighted	0.01	0.04	0.8
Intracranial Volume	470	MR Egger	0.09	0.11	0.
Intracranial Volume	470	Weighted median	0.02	0.05	0.7
Intracranial Volume	470	Weighted mode	0.01	0.10	0.9
Total Brain Volume	470	Inverse variance weighted	-0.03	0.04	0.4
Total Brain Volume	470	MR Egger	-0.01	0.11	0.9
Total Brain Volume	470	Weighted median	-0.05	0.05	0.3
Total Brain Volume	470	Weighted mode	-0.03	0.09	0.8
Grey Matter Volume	470	Inverse variance weighted	-0.07	0.04	0.06
Grey Matter Volume	470	MR Egger	-0.03	0.11	0.8
Grey Matter Volume	470	Weighted median	-0.09	0.05	0.06
Grey Matter Volume	470	Weighted mode	-0.07	0.09	0.5
Cortical Surface Area	470	Inverse variance weighted	-0.11	0.04	0.004
Cortical Surface Area	470	MR Egger	-0.04	0.11	0.7
Cortical Surface Area	470	Weighted median	-0.10	0.05	0.05
Cortical Surface Area	470	Weighted mode	-0.02	0.09	0.8
White Matter Volume	470	Inverse variance weighted	0.03	0.04	0.3
White Matter Volume	470	MR Egger	0.01	0.11	0.9
White Matter Volume	470	Weighted median	0.07	0.05	0.2
White Matter Volume	470	Weighted mode	0.13	0.12	0.3
Hippocampus	470	Inverse variance weighted	0.00	0.04	1.0
Hippocampus	470	MR Egger	0.00	0.11	1.0
Hippocampus	470	Weighted median	0.04	0.05	0.4
Hippocampus	470	Weighted mode	0.02	0.09	0.8
Amygdala	470	Inverse variance weighted	0.19	0.04	1.82E-07
Amygdala	470	MR Egger	0.04	0.11	0.7
Amygdala	470	Weighted median	0.14	0.05	0.005
Amygdala	470	Weighted mode	0.11	0.10	0.2
Thalamus	470	Inverse variance weighted	0.00	0.04	0.9
Thalamus	470	MR Egger	-0.09	0.11	0.4
Thalamus	470	Weighted median	-0.10	0.05	0.05
Thalamus	470	Weighted mode	-0.20	0.12	0.09

Supplemental Table 12: MR Egger Intercepts and Cochran's Q Statistics for Total Effect of Childhood Adiposity on Midlife Brain Traits when Outcomes Adjusted for Total Brain Volume Instead of Intracranial Volume

Outcome	MR Egger Intercept	p	Cochran's Q	p
Grey Matter Volume	0.000	0.8	429	<0.001
Cortical Surface Area	-0.002	0.2	527	<0.001
White Matter Volume	0.000	0.8	429	<0.001
Hippocampus	-0.001	0.4	390	<0.001
Amygdala	0.000	0.7	323	<0.001
Thalamus	-0.001	0.7	450	<0.001

Supplemental Table 13: Sensitivity Analyses for Total Effect of Childhood Adiposity on Midlife Brain Traits when Outcomes Adjusted for Total Brain Volume Instead of Intracranial Volume

Outcome	No. SNPs	Method	Beta	SE	p
Grey Matter Volume	266	Inverse variance weighted	-0.01	0.04	0.7
Grey Matter Volume	266	MR Egger	-0.03	0.10	0.7
Grey Matter Volume	266	Weighted median	-0.05	0.06	0.4
Grey Matter Volume	266	Weighted mode	-0.10	0.09	0.3
Cortical Surface Area	266	Inverse variance weighted	-0.08	0.05	0.1
Cortical Surface Area	266	MR Egger	0.04	0.11	0.7
Cortical Surface Area	266	Weighted median	-0.08	0.07	0.3
Cortical Surface Area	266	Weighted mode	-0.05	0.12	0.6
White Matter Volume	266	Inverse variance weighted	0.01	0.04	0.7
White Matter Volume	266	MR Egger	0.03	0.10	0.7
White Matter Volume	266	Weighted median	0.05	0.06	0.4
White Matter Volume	266	Weighted mode	0.10	0.09	0.3
Hippocampus	266	Inverse variance weighted	-0.08	0.04	0.07
Hippocampus	266	MR Egger	0.00	0.10	1.0
Hippocampus	266	Weighted median	-0.09	0.07	0.2
Hippocampus	266	Weighted mode	-0.05	0.10	0.5
Amygdala	266	Inverse variance weighted	-0.01	0.04	0.8
Amygdala	266	MR Egger	0.02	0.09	0.9
Amygdala	266	Weighted median	-0.04	0.07	0.6
Amygdala	266	Weighted mode	-0.07	0.10	0.5
Thalamus	266	Inverse variance weighted	-0.21	0.05	1.07E-05
Thalamus	266	MR Egger	-0.17	0.11	0.1
Thalamus	266	Weighted median	-0.19	0.07	0.006
Thalamus	266	Weighted mode	-0.11	0.09	0.2

Supplemental Table 14: MR Egger Intercepts and Cochran's Q Statistics for Total Effect of Adult Adiposity on Midlife Brain Traits when Outcomes Adjusted for Total Brain Volume Instead of Intracranial Volume

Outcome	MR Egger Intercept	p	Cochran's Q	p
Grey Matter Volume	-0.001	0.5	819	<0.001
Cortical Surface Area	-0.001	0.4	968	<0.001
White Matter Volume	0.001	0.5	819	<0.001
Hippocampus	0.002	0.2	688	<0.001
Amygdala	-0.001	0.7	626	<0.001
Thalamus	0.002	0.1	808	<0.001

Supplemental Table 15: Sensitivity Analyses for Total Effect of Adult Adiposity on Midlife Brain Traits when Outcomes Adjusted for Total Brain Volume Instead of Intracranial Volume

Outcome	NoSNPs	Method	Beta	SE	p
Grey Matter Volume	470	Inverse variance weighted	-0.09	0.04	0.0
Grey Matter Volume	470	MR Egger	-0.01	0.11	0.9
Grey Matter Volume	470	Weighted median	-0.13	0.05	0.008
Grey Matter Volume	470	Weighted mode	-0.18	0.10	0.08
Cortical Surface Area	470	Inverse variance weighted	-0.17	0.04	7.03E-05
Cortical Surface Area	470	MR Egger	-0.08	0.13	0.5
Cortical Surface Area	470	Weighted median	-0.13	0.06	0.03
Cortical Surface Area	470	Weighted mode	-0.15	0.12	0.2
White Matter Volume	470	Inverse variance weighted	0.09	0.04	0.02
White Matter Volume	470	MR Egger	0.01	0.11	0.9
White Matter Volume	470	Weighted median	0.13	0.05	0.009
White Matter Volume	470	Weighted mode	0.18	0.10	0.07
Hippocampus	470	Inverse variance weighted	0.04	0.04	0.4
Hippocampus	470	MR Egger	-0.10	0.11	0.4
Hippocampus	470	Weighted median	0.00	0.06	1.0
Hippocampus	470	Weighted mode	-0.04	0.11	0.7
Amygdala	470	Inverse variance weighted	-0.01	0.04	0.7
Amygdala	470	MR Egger	0.03	0.11	0.8
Amygdala	470	Weighted median	-0.08	0.05	0.1
Amygdala	470	Weighted mode	-0.17	0.12	0.2
Thalamus	470	Inverse variance weighted	-0.08	0.04	0.05
Thalamus	470	MR Egger	-0.25	0.12	0.04
Thalamus	470	Weighted median	-0.14	0.06	0.01
Thalamus	470	Weighted mode	-0.14	0.11	0.2

Supplemental Table 16: MVMR Results Demonstrating Effects of Childhood and Adult Adiposity on Midlife Brain Traits Without Any Form of Head Size Adjustment

Outcome	Age	Beta	SE	p
Intracranial Volume	Childhood	0.22	0.061	3.43E-04
Intracranial Volume	Adult	-0.10	0.05	0.052
Total Volume	Childhood	0.14	0.06	0.02
Total Volume	Adult	-0.10	0.05	0.053
Grey Matter Volume	Childhood	0.16	0.06	0.006
Grey Matter Volume	Adult	-0.15	0.05	0.003
Cortical Surface Area	Childhood	0.16	0.06	0.01
Cortical Surface Area	Adult	-0.19	0.05	4.06E-04
Cortical Thickness	Childhood	0.06	0.06	0.3
Cortical Thickness	Adult	0.00	0.05	1.0
White Matter Volume	Childhood	0.10	0.06	0.08
White Matter Volume	Adult	-0.04	0.05	0.4
White Matter Hyperintensities	Childhood	-0.20	0.06	9.54E-04
White Matter Hyperintensities	Adult	0.29	0.05	1.36E-08
Amygdala	Childhood	0.06	0.06	0.3
Amygdala	Adult	-0.07	0.05	0.2
Hippocampus	Childhood	-0.03	0.06	0.6
Hippocampus	Adult	0.03	0.05	0.5
Thalamus	Childhood	-0.01	0.06	0.9
Thalamus	Adult	-0.07	0.05	0.2

Supplemental Table 17: MVMR Results Demonstrating Effects of Childhood and Adult Adiposity on Midlife Brain Traits when Outcomes Adjusted for Total Brain Volume Instead of Intracranial Volume

Outcome	Age	Beta	SE	p
Grey Matter Volume	Childhood	0.05	0.06	0.4
Grey Matter Volume	Adult	-0.14	0.05	0.01
Cortical Surface Area	Childhood	0.04	0.07	0.6
Cortical Surface Area	Adult	-0.20	0.0	6.79E-04
White Matter Volume	Childhood	-0.05	0.06	0.4
White Matter Volume	Adult	0.14	0.05	0.01
Amygdala	Childhood	-0.01	0.06	0.9
Amygdala	Adult	-0.01	0.05	0.8
Hippocampus	Childhood	-0.16	0.06	0.009
Hippocampus	Adult	0.13	0.05	0.016
Thalamus	Childhood	-0.23	0.07	6.13E-04
Thalamus	Adult	0.04	0.06	0.5

Supplemental Table 18: MVMR Results Demonstrating Effects of Birthweight and Childhood Adiposity on Midlife Brain Traits

Outcome	Age	Beta	SE	p
Total Volume	Birthweight	-0.04	0.06	0.6
Total Volume	Childhood	-0.20	0.04	3.19E-06
Grey Matter Volume	Birthweight	-0.12	0.05	0.027
Grey Matter Volume	Childhood	-0.17	0.04	1.58E-05
Cortical Surface Area	Birthweight	-0.07	0.07	0.4
Cortical Surface Area	Childhood	-0.26	0.05	1.75E-06
Cortical Thickness	Birthweight	-0.01	0.06	0.8
Cortical Thickness	Childhood	0.07	0.04	0.1
White Matter Volume	Birthweight	0.08	0.07	0.2
White Matter Volume	Childhood	-0.15	0.05	0.005
White Matter Hyperintensities	Birthweight	0.08	0.07	0.2
White Matter Hyperintensities	Childhood	0.01	0.05	0.9
Amygdala	Birthweight	-0.26	0.06	4.69E-05
Amygdala	Childhood	-0.05	0.05	0.3
Hippocampus	Birthweight	-0.21	0.06	0.001
Hippocampus	Childhood	-0.14	0.05	0.002
Thalamus	Birthweight	-0.08	0.07	0.2
Thalamus	Childhood	-0.30	0.05	4.69E-09

SUPPLEMENTAL FIGURES

Supplemental Figure 1: Forest Plots for Total and Direct Effects of Childhood and Adult Adiposity on Midlife Brain Traits Without Adjustment for Intracranial Volume

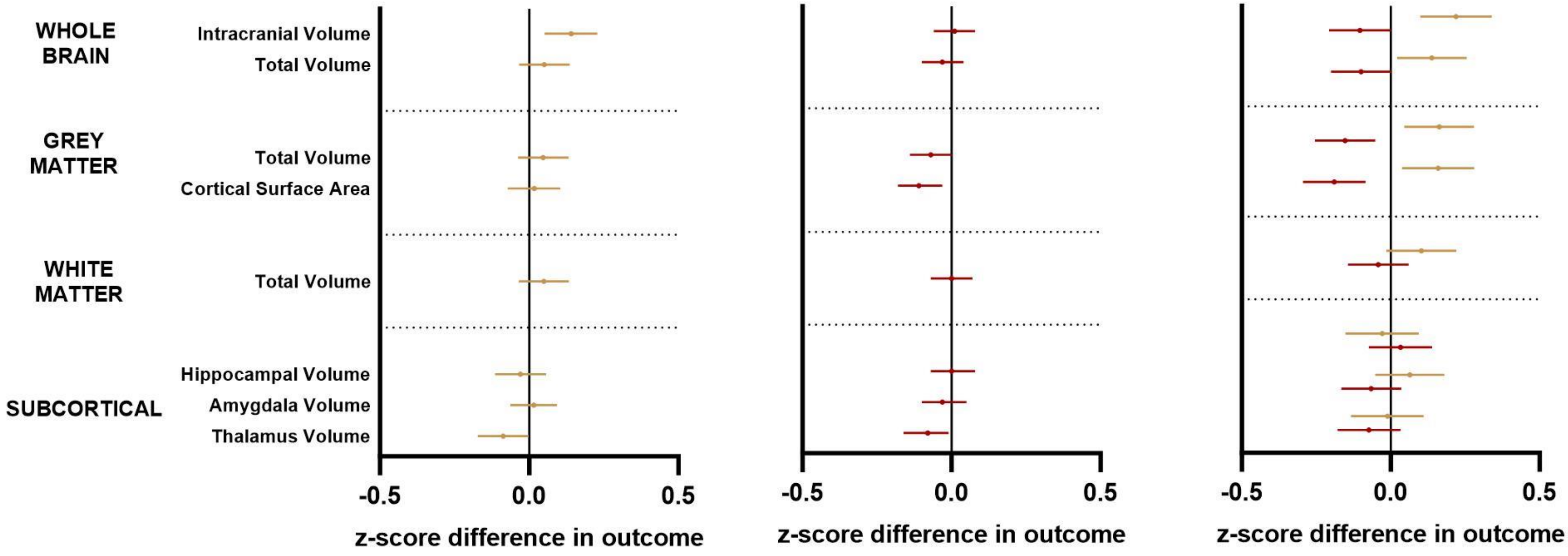

Supplemental Figure 2: Forest Plots for Total Effects of Childhood and Adult Adiposity on Midlife Brain Traits when Outcomes Adjusted for Total Brain Volume

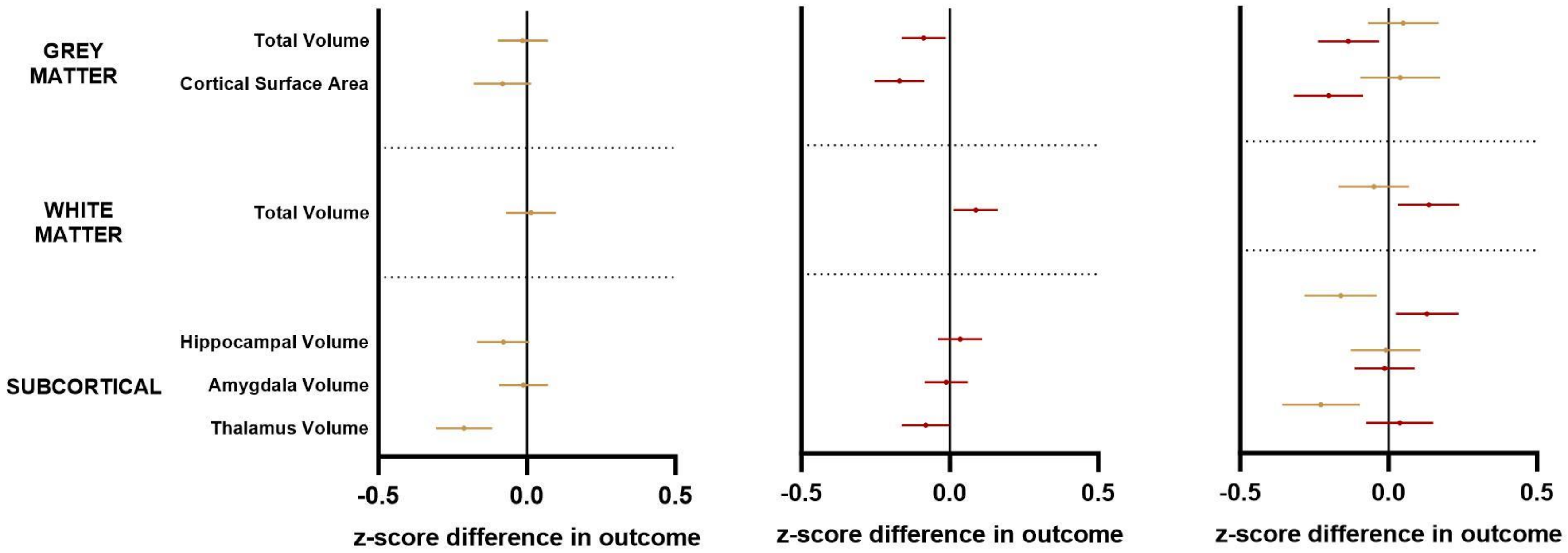

Supplemental Figure 3: Funnel Plots Demonstrating Balance of Pleiotropic Effects for Each Brain Trait in Main Analysis Across all Childhood Instruments

Total Brain Volume

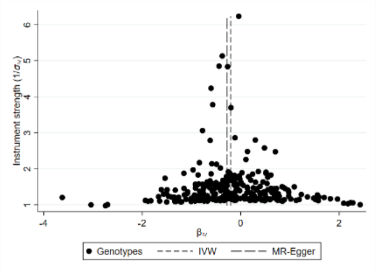

Grey Matter Volume

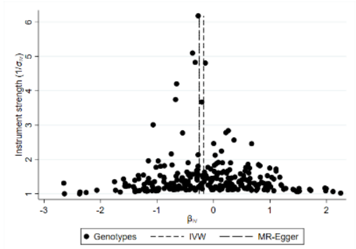

Cortical Surface Area

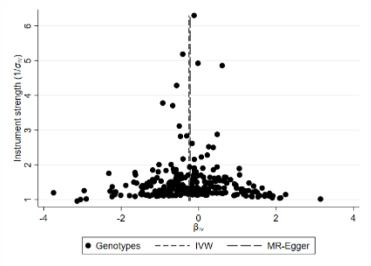

Cortical Thickness

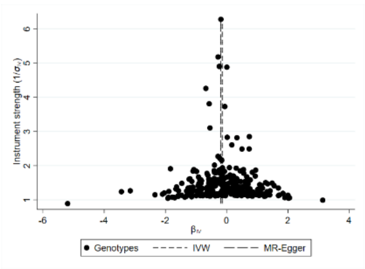

White Matter Volume

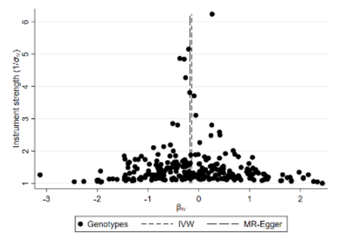

White Matter Hyperintensities

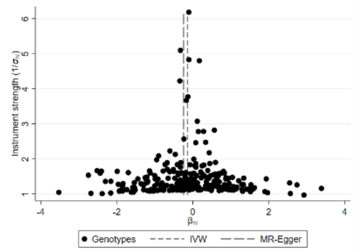

Hippocampus Volume

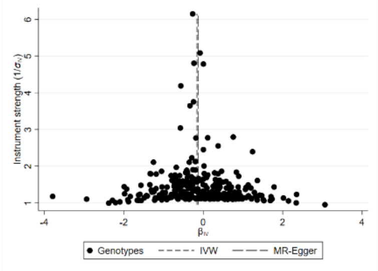

Amygdala Volume

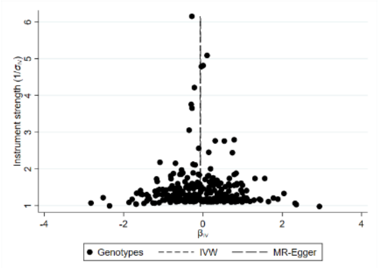

Thalamus Volume

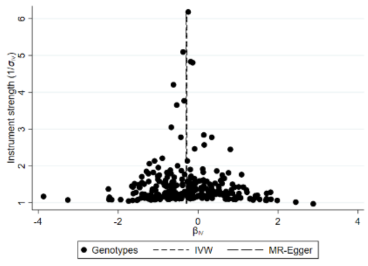

Supplemental Figure 4: Funnel Plots Demonstrating Balance of Pleiotropic Effects for Each Brain Trait in Main Analysis Across all Adult Instruments

Total Brain Volume

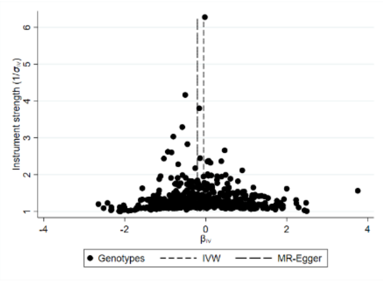

Grey Matter Volume

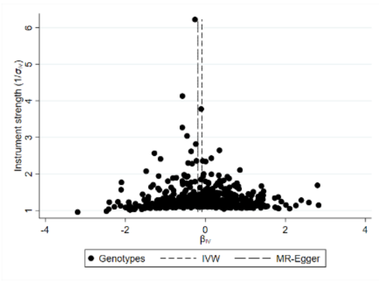

Cortical Surface Area

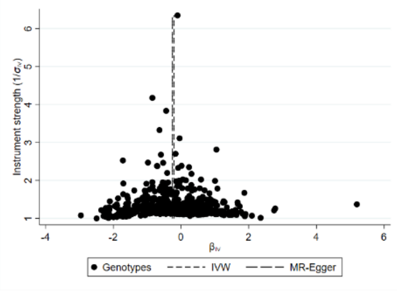

Cortical Thickness

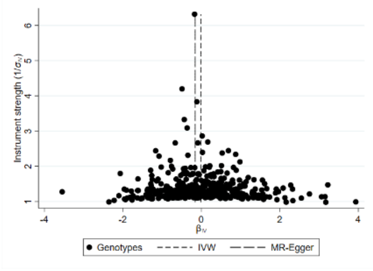

White Matter Volume

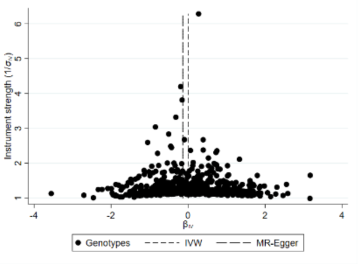

White Matter Hyperintensities

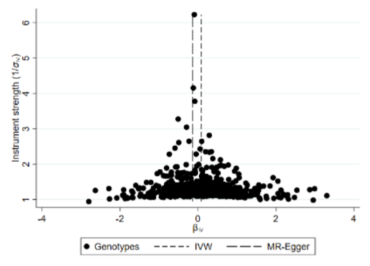

Hippocampus Volume

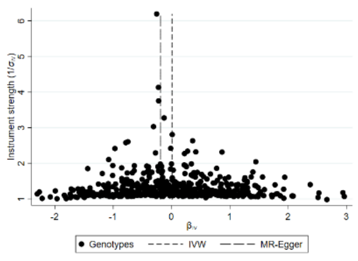

Amygdala Volume

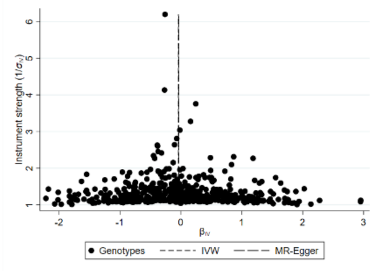

Thalamus Volume

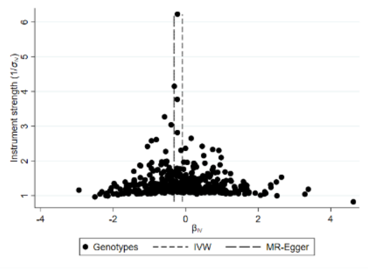

Supplemental Figure 5: Leave-One-Out Analyses to Test for Undue Influence of Single Genetic Variants Which May Potentially Impact Main Analysis of Childhood Total Effects

Total Volume

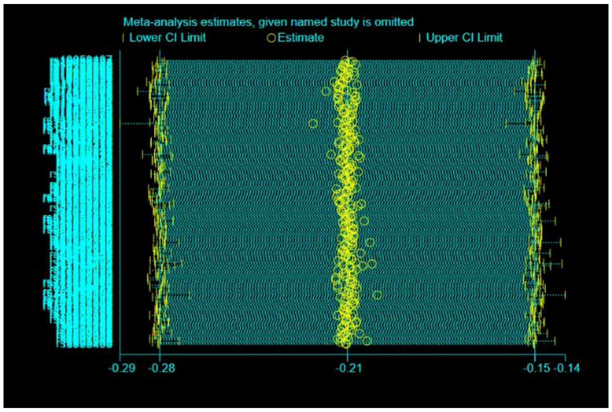

Grey Matter Volume

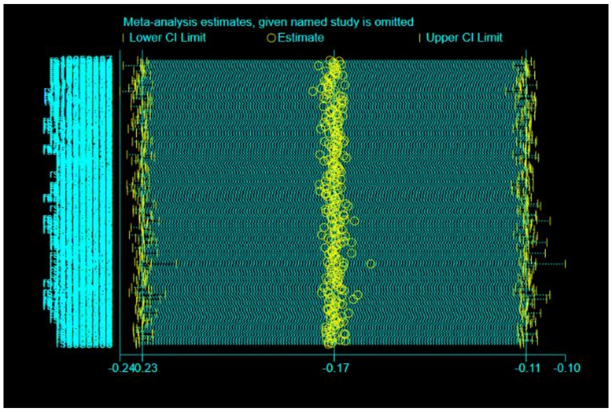

Cortical Surface Area

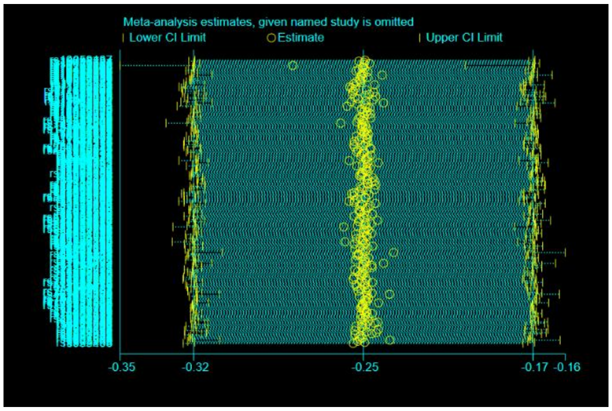

Cortical Thickness

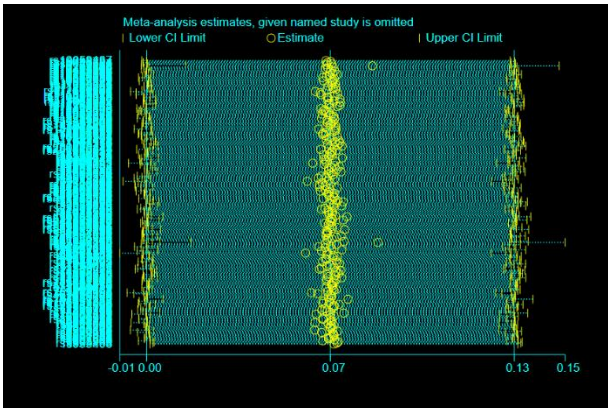

White Matter Volume

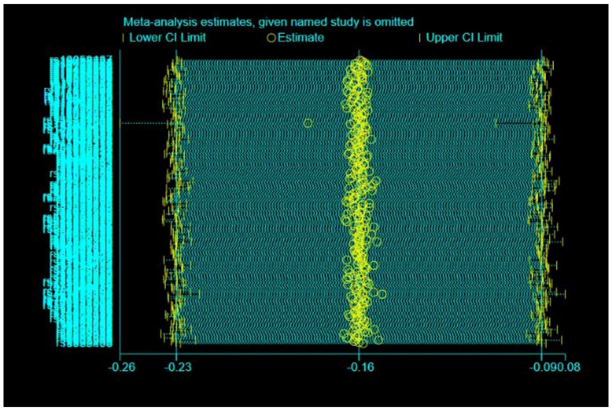

White Matter Hyperintensities

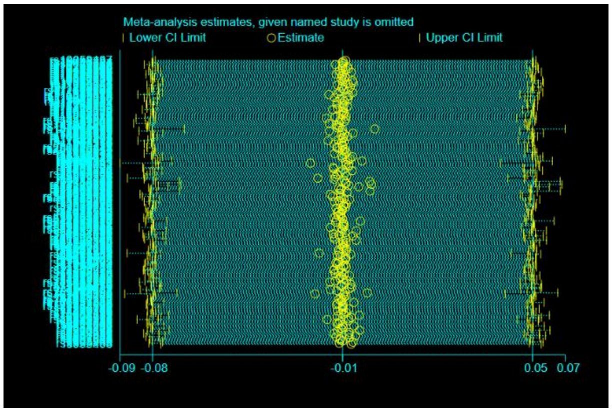

Amygdala

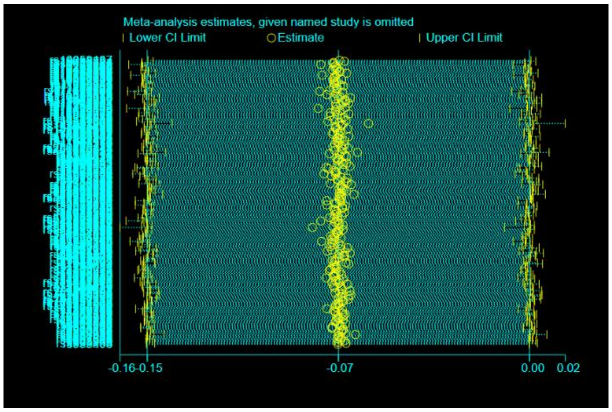

Hippocampus

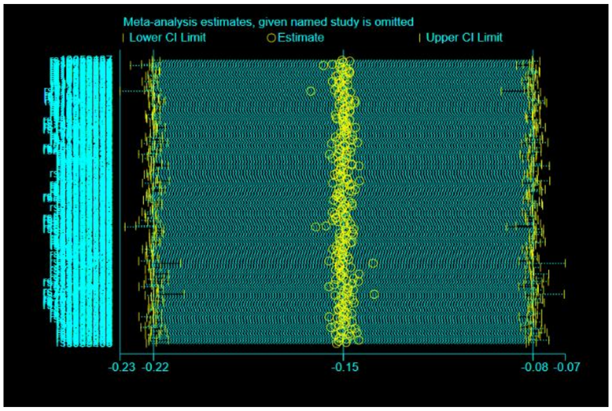

Thalamus

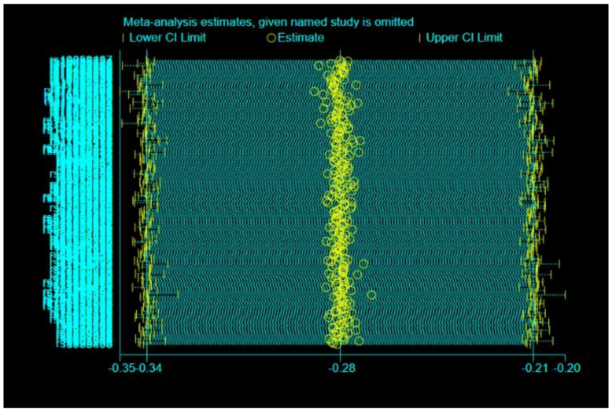

Supplemental Figure 6: Leave-One-Out Analyses to Test for Undue Influence of Single Genetic Variants Which May Potentially Impact Main Analysis of Adult Total Effects

Total Brain Volume

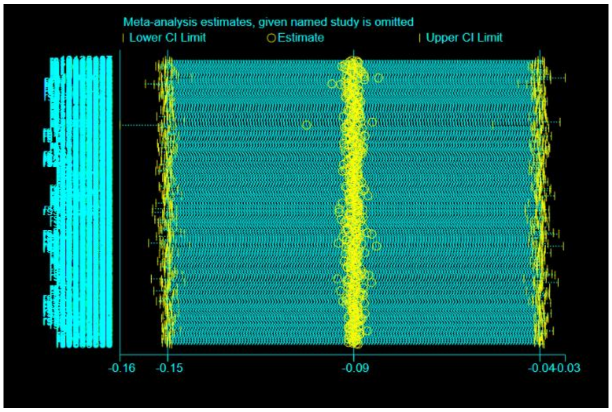

Grey Matter Volume

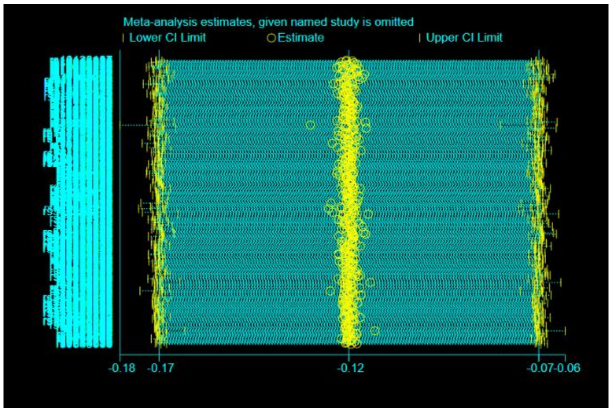

Cortical Surface Area

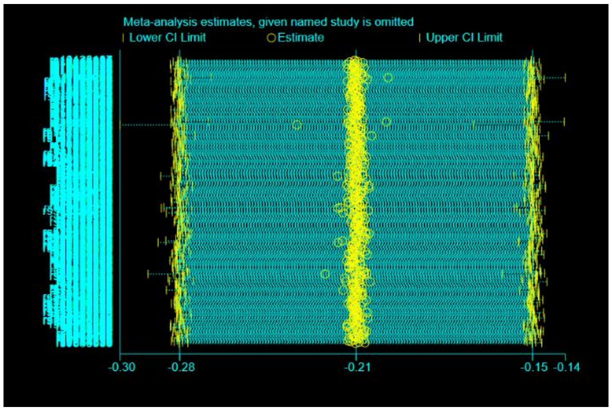

Cortical Thickness

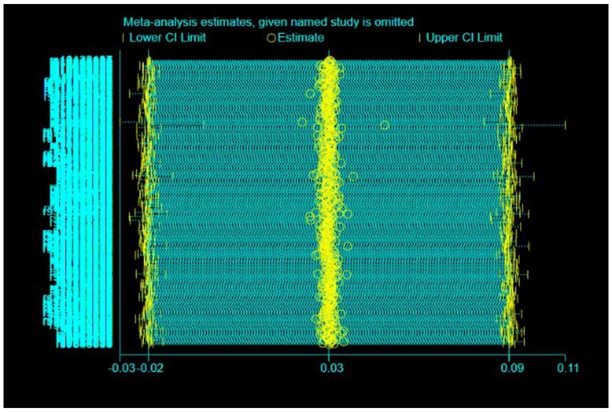

White Matter Volume

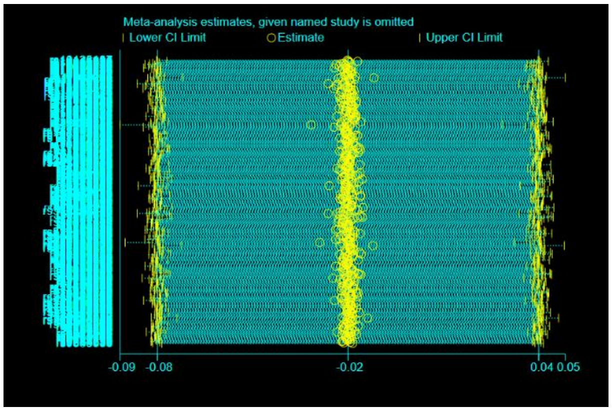

White Matter Hyperintensities

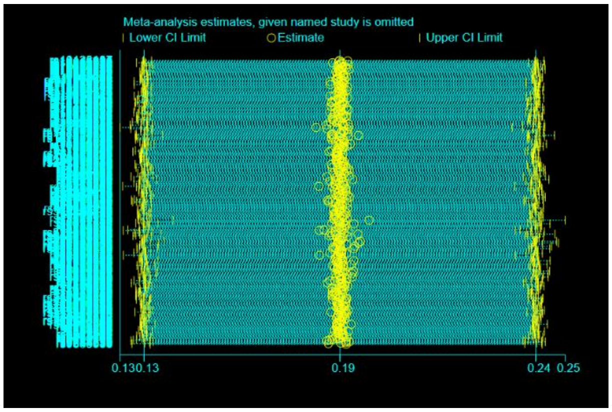

Amygdala

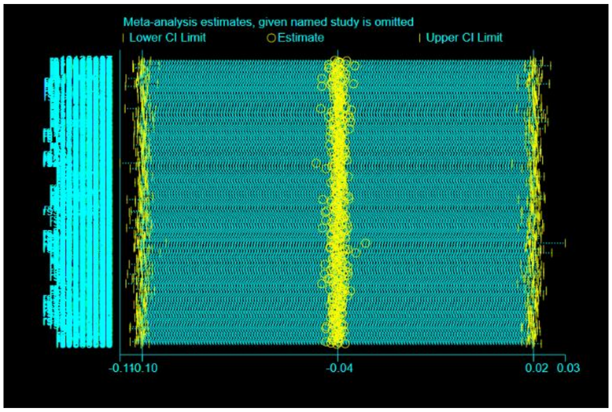

Hippocampus

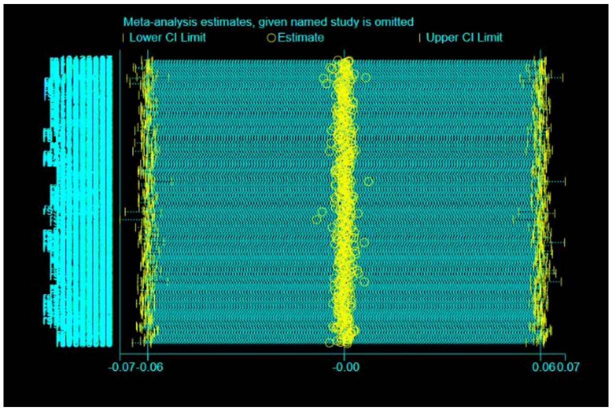

Thalamus

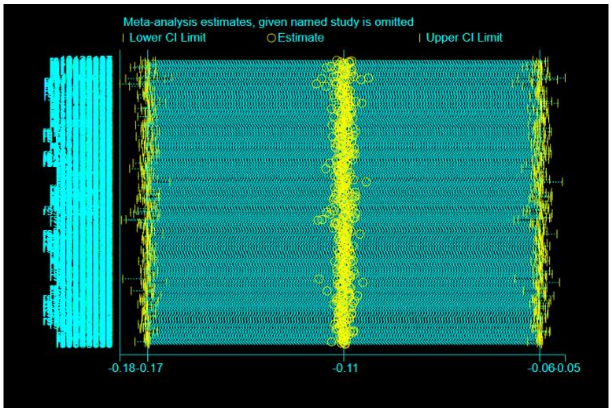

Supplement: awae198_Supplementary_Data [file awae198_supplementary_data.pdf]
